# A high-throughput phenotyping procedure for evaluation of antixenosis against common cutworm at early seedling stage in soybean

**DOI:** 10.1186/s13007-017-0215-1

**Published:** 2017-08-07

**Authors:** Guangnan Xing, Kai Liu, Junyi Gai

**Affiliations:** Soybean Research Institute/National Center for Soybean Improvement/MOA Key Laboratory for Biology and Genetic Improvement of Soybean (General)/State Key Laboratory for Crop Genetics and Germplasm Enhancement/Jiangsu Collaborative Innovation Center for Modern Crop Production, Nanjing Agricultural University, Nanjing, 210095 Jiangsu People’s Republic of China

**Keywords:** Soybean, Common cutworm (CCW), High-throughput phenotyping, Antixenosis

## Abstract

**Background:**

Common cutworm (CCW; *Spodoptera litura* Fabricius) is a major leaf-feeding pest of soybean in Asia. The previous methods of measuring antixenosis against CCW using adult plant under field or net-room conditions were time-consuming, labor-intensive and precision-inferior. To solve the problems, this study aimed at (i) establishing a high-throughput phenotyping method for evaluating antixenosis against CCW at early seedling stage, (ii) using the procedure to evaluate the antixenosis of an insect-resistant versus -susceptible germplasm population (IRSGP), (iii) validating the proposed method through comparing the results with the historical phenotypic data and phenotyping-genotyping consistency data using PAV (presence/absence variation) markers linked with the identified loci *CCW*-*1* and *CCW*-*2*, (iv) and evaluating the efficiency of the novel method through comparisons to the previous methods.

**Results:**

A dynamic and efficient evaluation procedure characterized with using V1 stage soybean seedlings infested with third-instar larvae in a micro-net-room in greenhouse with damaged leaf percentage (DLP) as indicator was established and designated V1TMD method. The middle term testing stage is the best dates for identifying resistant and susceptible accessions. The results from the V1TMD method were relatively stable, precise and accurate in comparison with the previous method with the detected most resistant and susceptible accessions consistent to the previous results. The DLP values differentiated obviously to coincide with the resistant and susceptible alleles of the PAV markers Gm07PAV0595 and Gm07PAV0389 tightly linked to the two resistance-related loci, *CCW*-*1* and *CCW*-*2*, respectively, in IRSGP. Thus V1TMD is a high-throughput phenotyping method with its estimated efficiency 12 times and period shortening 4 times of those of the previous method.

**Conclusion:**

A dynamic and efficient V1TMD method for testing antixenosis against CCW was established, with highly resistant and highly susceptible accessions as standard checks and DLP as indicator. The method is remarkably quick, highly reproducible, and capable of testing large samples, therefore, is a high-throughput phenotyping method.

**Electronic supplementary material:**

The online version of this article (doi:10.1186/s13007-017-0215-1) contains supplementary material, which is available to authorized users.

## Background

Soybean (*Glycine max* [L.] Merr.) is one of the important crops in the world for its high contents of edible protein and oil [1]. Common cutworm (CCW; *Spodoptera litura* Fabricius) is a major leaf-feeding pest of soybean because of its polyphagous characteristic and rapid multiplication [2]. Until now, the major way to prevent pests is the frequent use of insecticides, thus applying resistant accessions would contribute to integrated pest management in a sustainable and environment-friendly manner [3]. The insect-resistance has been incorporated into the breeding programs [4]. Among the plant introductions from USDA Soybean Germplasm Collection, PI171451, PI227687 and PI229358 were used in breeding for insect resistance because they provided multiple resistances to several insects, including Mexican bean beetle (*Epilachna varivestis* Mulsant), soybean looper (*Pseudoplusia includes* Walker), velvetbean caterpillar (*Anticarsia gemmatalis*), and corn earworm (*Helicoverpa zea* Boddie) [5–8]. There is a collection of insect-resistant versus susceptible soybean germplasm in National Center for Soybean Improvement of China, mainly from the evaluation of domestic and foreign germplasm [9–11].

Host plant resistance could be classified as antixenosis (or nonpreference), antibiosis and tolerance [12, 13]. Antixenosis is known as non-preference and can repel insects from the host plant because of no or less attractant components such as volatile organic compounds, colors and morphologies of plant surfaces [14]. Antibiosis leads to insect abnormal performances such as physiological or developmental detriment due to toxin chemicals or secondary metabolites which are produced by the resistant plants [7]. Tolerance is the plant’s ability to respond to insect damage through repair and reproduction to reduce the impact of insect on the plant itself [15].

The method of resistance evaluation is the basis of accelerating insect resistance breeding processes. The evaluation methods of antibiosis [11, 16] and antixenosis [9, 10, 13, 17, 18] against CCW for adult plants have been established in soybean, which mainly include natural field infestation [9, 17, 18], artificial infestation in net-room [10, 13] and paired-comparison test of the feeding preferences in Petri dish [19]. In natural field infestation, the plant materials were randomly grown in field with certain replications, and the evaluation indicators were DLP (damaged leaf percentage) [9, 17] or CCW larvae densities [18] under field infestation, respectively. In artificial infestation in netroom, the materials were organized in a randomized complete block design hill plot experiment with hill plot spacing of 0.6 × 0.7 m^2^ in netroom free from invasion of other insects, and the hill plots were infested with CCW third instar larvae with DLP as resistant indicator [13]. In paired-comparison test of the feeding preferences in Petri dish, a standard (check) and a test leaflet segments were pairwisely laid with the abaxial side facing up on the filter paper in a Petri dish (90 mm in diameter, 20 mm in depth), and a third-instar CCW larvae was placed between the leaflet segments in an air-conditioned room; 14 h later, the visual defoliation ratings were assessed for the two leaflet segments [19]. Under natural field infestation, the non-biological (such as extreme temperature, rainstorm, contingency disaster environment) and biological factors (including the harm of natural enemies and infectious disease) could not be avoided, and even so sometimes under net-room artificial infestation. Compared with the antixenosis of living plants to CCW, antixenosis bioassay of detached leaves was affected by their fresh degree [19]. There were many other leaf-feeding insects that ate the leaves of soybean in the field, which affected the evaluation of antixenosis against CCW. The CCW larvae densities in the field varied remarkably from year to year, which was mainly caused by different weather conditions [9, 18]. When plants reached adult stage, many leaves in each plot usually overlapped each other, resulting in a difficult estimation of the DLP for each accession. Meanwhile the large number of leaves needed a large amount of CCW larvae in the previous method. If the inoculated larvae was not enough or the environment did not help damage, the average DLP was still low until the end of the test, so that the resistant accessions could not be distinguished from the susceptible ones. Since the previous evaluation methods take much space and manpower, it could not fit testing a large scale germplasm population, or in other words, the shortcomings of the previous method lies in larger experiment error, longer time consuming, more labor consumption, higher cost and lower efficiency.

With the development of high-throughput sequencing technologies, it is easy to obtain the plant genotype data, but it is relatively difficult to gain the accurate phenotype data [20]. So to match the requirements for high-resolution linkage mapping, genome-wide association studies and establishment of genomic selection models in plants, an accurate and high-throughput phenotyping strategy is needed urgently [21]. The objective of modern phenotyping is to improve the accuracy and throughput of phenotypic measurement with lowering costs and decreasing labor consumption [22]. Therefore, an increasing attention has been paid to high-throughput phenotyping method.

The CCW resistance had been mapped to two linked major loci (*CCW*-*1* and *CCW*-*2*) in the linkage group M [23, 24]. The QTL-M was previously detected for both antixenosis and antibiosis against corn earworm in Cobb × PI229358 and Cobb × PI171451 mapping populations [14, 25–27], which is identical to *CCW*-*1*, but the *CCW*-*2* was not detected. With the completion of reference genome sequencing, studies on the genome sequence differences have been become a hot focus [28, 29]. A new molecular marker system, presence/absence variation (PAV) of large fragment genomic sequences, shows potential because of its PCR-based convenience and distinct band recognization. Its application in soybeans has been widely reported [28].

The present study aimed at (i) establishing a high-throughput phenotyping method for evaluating antixenosis against CCW at young seedling stage, (ii) using the procedure to evaluate the antixenosis of an insect-resistant versus -susceptible germplasm population (IRSGP), (iii) validating the proposed method through comparing the results with the historical phenotypic data and phenotyping-genotyping consistency data using PAV (presence/absence variation) markers linked with the identified loci *CCW*-*1* and *CCW*-*2*, (iv) and evaluating the efficiency of the novel method through comparisons to the previous methods.

## Methods

### Plant materials

A representative sample of the insect-resistant versus -susceptible germplasm population (IRSGP) obtained from the National Center for Soybean Improvement of China was used to test the efficiency of the improved evaluation method for antixenosis against CCW (Experiment 1). The IRSGP composes of 76 highly resistant versus susceptible accessions, which was screened out from 6724 domestic and foreign germplasm resources for resistance to comprehensive leaf-feeding insects under natural field infestations [9], artificial infestation in net-room [10], and antibiosis evaluation in laboratory [11]. The international well-known resistant source PI227687 [5] and Lamar [30] are included in the IRSGP as insect resistant checks.

### CCW larvae reared for infesting soybeans

The CCW pupa stock was obtained from the Entomology Laboratory of Nanjing Agriculture University, and was cultured to obtain large number of eggs. The CCW population was reared in the insectarium at 28 ± 1 °C under 16 h light/8 h dark photoperiod rhythm [16]. The third-instar larvae with uniform size were used for evaluating the antixenosis against CCW in all experiments conducted.

### Facilities and procedures for evaluating antixenosis against CCW

**Fig. 1:**
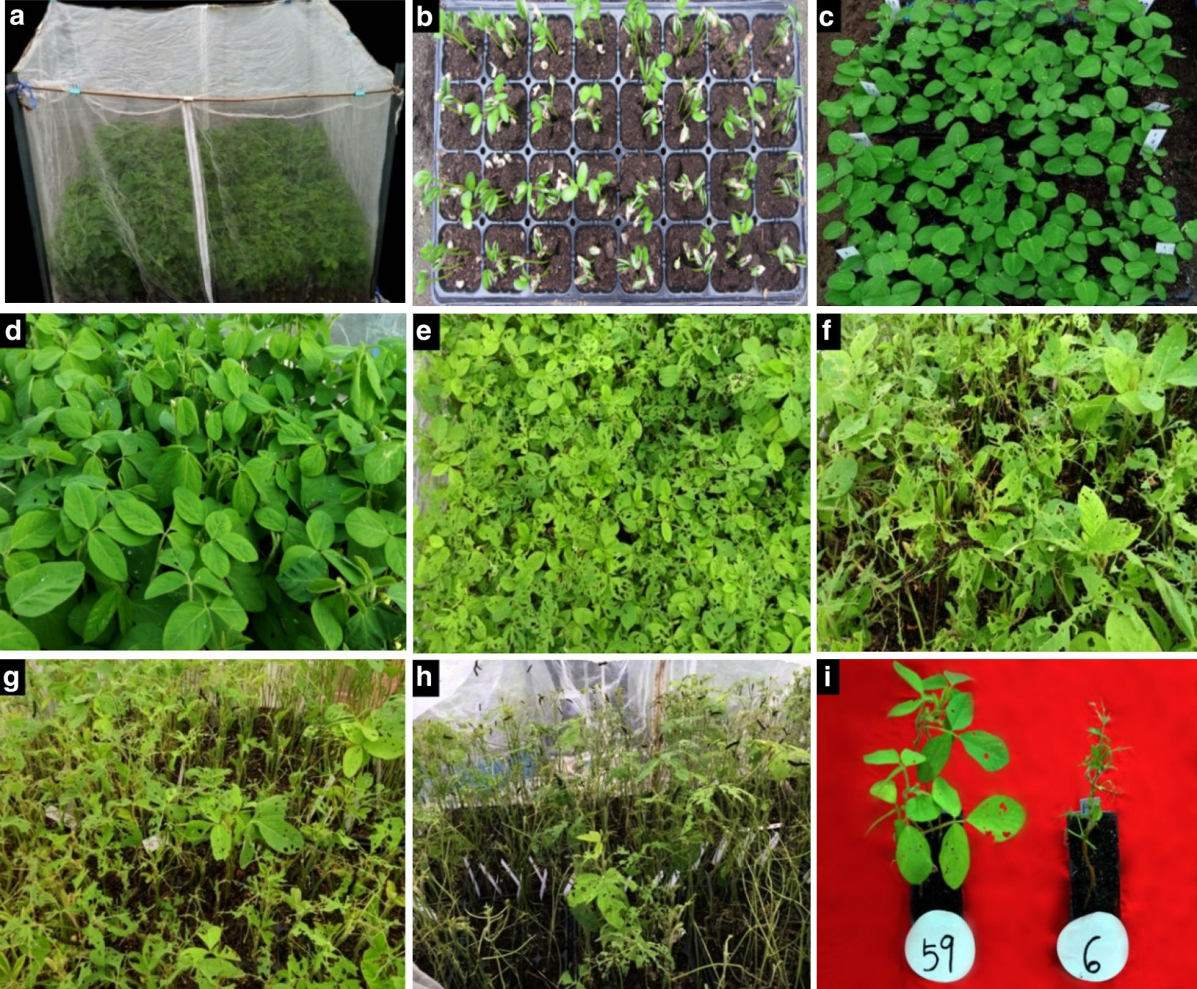
Major technical procedures of the V1TMD method for evaluation of antixenosis against common cutworm in soybean seedlings. **a** The micro-netroom in greenhouse; **b** soybean seedlings at VE stage in seed nursery tray; **c** soybean seedlings at VC stage in seed nursery tray; **d** soybean seedlings at V1 stage, when canopy structure has formed and artificial infestation has started; **e** the average DLP of whole accessions is about 35% after artificial infestation; **f** the average DLP of whole accessions is about 50%; **g** the average DLP of whole accessions is about 70%; **h** the average DLP of whole accessions was about 80%; **i** the photo of highly resistant accession (59, Lamar) and highly susceptible accession (6, MYBMD)

The evaluation facilities of V1TMD method for antixenosis against CCW include an insecticide–free micro-net-room in greenhouse and 32-hole small seed nursery tray (54, 28 and 6 cm in length, width and height, respectively) (Fig. 1a). The size of the micro-net-room is varied according to the number of seed nursery trays. For example, a micro-net-room of about 1.52 m^2^ is needed for 10 seed nursery trays. Six seeds were sown in each hole of the 32-hole seed nursery tray. At about 10 days after sowing (Fig. 1b) the seedlings reached to VE (emergence) stage [31], then they were thinned to two plants in each hole (Fig. 1c) at VC (cotyledon) stage [31]. The artificial infestations were initiated (Fig. 1d) when the leaves of the seedlings in a nursery tray formed a connected canopy at V1 (first trifoliolate) stage that allowing CCW larvae to move freely for choosing leaves among different accessions. Two third-instar CCW larvae with visually uniform size were applied to leaves of each accession using small, natural-hair paint brush. After artificial infestation for 3 days, the DLP of each accession was investigated successively for several times until the average DLP of accessions over 80%. The average DLP of whole accessions was about 35% after artificial infestation for 5–7 days, at this time, the susceptible accessions were easily to be identified (Fig. 1e). The best evaluation time for distinguishing and grading antixenosis against CCW was at the time when the average DLP of whole materials reaching 50–70% (Fig. 1f, g) because a wide range of DLP among accessions was shown (Table 1). In addition, when the average DLP of whole materials reached more than 80% (Fig. 1h), there were only highly resistant accessions with DLP below 40% and therefore it was suitable time for identifying highly resistant accessions (Fig. 1i). Thus from the dynamic evaluation process, highly resistant and highly susceptible accessions could be easily identified while at late (DLP > 80%) and early (DLP < 35%) stage, respectively, but for comparisons among all accessions the suitable time should be at the mid-term (DLP around 50–70%).

**Table 1:** Dynamic frequency distribution and descriptive statistics of DLP in IRSGP

Indicator and date	Class mid-point of DLP (%)										Mean (%)	Range (%)	*F*	*h* ^2^ (%)	*GCV* (%)	*CV* (%)
	10	20	30	40	50	60	70	80	90	100						
DLP1-3	46	23	7								14	0–30	2.2**	54.3	39.8	69.7
DLP1-4	18	40	16	2							19	3–36	2.8**	64.3	33.3	49.4
DLP1-5	9	23	28	11	4	1					27	5–55	3.7**	72.6	35.5	42.2
DLP1-6	3	11	27	24	8	3					33	5–64	4.6**	78.3	32.0	33.4
DLP1-7	3	9	16	23	18	6	1				37	6–71	5.7**	82.4	32.8	30.1
DLP1-8	2	2	14	25	19	11	3				42	6–74	5.5**	82.0	29.1	27.1
DLP1-9	2	1	13	25	18	15	2				44	5–71	5.5**	81.7	27.8	25.7
*DLP1-10*	2	1	7	16	20	18	10	2			50	5–80	6.6**	85.0	26.8	22.3
*DLP1-11*	1	1	1	10	16	18	24	3	2		59	9–93	6.0**	83.4	22.8	20.2
*DLP1-12*	1	1			2	14	24	22	11	1	72	14–100	6.4**	84.3	18.7	16.0
DLP1-13			1	1		1	9	22	28	14	84	25–100	6.9**	85.4	14.7	12.1
DLP1-14			1		1			10	30	34	92	34–100	7.4**	86.5	11.2	8.8
DLP1-15				1	1			2	14	58	95	36–100	10.8**	90.7	9.0	6.0
DLP2-3	40	27	6	3							15	0–44	2.8**	63.9	47.0	69.9
DLP2-4	9	26	21	10	6	4					28	1–64	4.3**	76.7	43.8	48.1
DLP2-5	4	8	13	24	11	9	5	2			41	3–79	4.2**	76.9	34.7	38.3
*DLP2-6*	3	3	11	10	17	13	7	9	3		51	5–88	9.8**	89.8	36.9	24.5
*DLP2-7*	2	1	3	8	15	16	12	10	8	1	60	5–96	9.6**	88.9	31.4	21.2
*DLP2-8*	2		2	2	12	16	13	13	11	5	67	5–99	9.6**	89.6	27.8	19.0
DLP2-9	1	1	2	1	3	11	16	18	13	10	75	8–100	10.7**	90.7	25.1	16.0
DLP2-10	1	1	1	1	2	2	13	19	20	16	80	9–100	10.4**	90.3	22.3	14.5
DLP2-11	1	1		1	1	1	3	11	33	24	87	9–100	13.9**	92.8	18.1	10.1

In “Indicator and date” column, “DLP” represents damaged leaf percentage, the number before “-” indicates the test serial and the number after”-” indicates the days after artificial infestation, for example, “DLP1-3” represents DLP after 3 days of infestation in Test 1; those in italics represent the best date for evaluation

*IRSGP* insect-resistant versus -susceptible germplasm population, *h* ^2^ heritability, *GCV* genotypic coefficient of variation, *CV* error coefficient of variation

** Represents significance at 0.01 probability level

### Indicator and criterion for evaluation of antixenosis against CCW

**Fig. 2:**
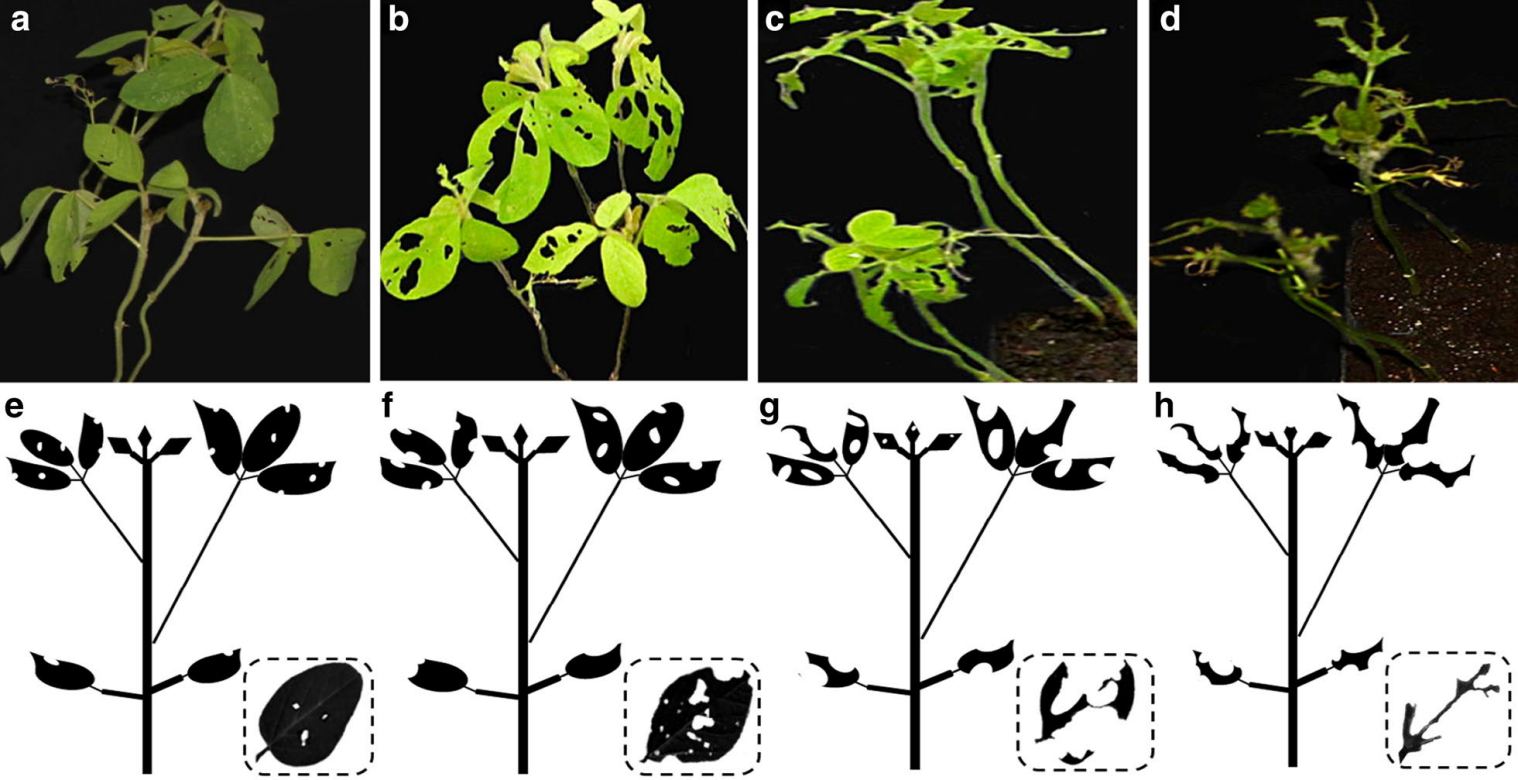
Examples of the visual defoliation rating. **a–d** The average DLP is about 10, 30, 70 and 90%, respectively, in a real photo. **e–h** The average DLP is also 10, 30, 70 and 90%, respectively, in the drown picture with a leaflet defoliation chart at the *lower right corner*

The damaged leaf percentage (DLP) is used as the antixenosis indicator to CCW. The minimum value of DLP was 0%, which represents that leaves were intact. In contrast, the maximum value of DLP was 100%, which represents that leaves were fully defoliated. Visual observation was used for the evaluation of DLP with an interval of 5% difference. The average DLP of a plant was roughly calculated from all leaflets (There are a total of 8 leaflets for a seedling plant at V2 stage, its DLP is easy to be estimated due to only fewer leaflets), and the average DLP of two plants per hole was used for an accession. Figure 2 shows roughly the evaluation criterion. When the average DLP is below 30%, the leaflets appear a normal shape but with small or big holes (Fig. 2a, b, e, f). However, when the average DLP is above 70%, the leaflets appear incomplete shape with large holes even only middle and lateral main veins remained (Fig. 2c, d, g, h). For a consistent and accurate evaluation of the DLP value, the observer should be skilled and should work through all the tests at least a whole replication for keeping out the personal biases.

From the above, the evaluation procedure characterized with using V1 stage of soybean seedlings infested with third-instar larvae in micro-net-room in greenhouse with damaged leaf percentage (DLP) as indicator is a novel dynamic and efficient evaluation procedure for antixenosis against CCW in soybean. Since the testing net-room size, testing time and testing expense are reduced, but the accuracy and precision are increased, we designated it as a high-throughput phenotyping method for evaluating antixenosis against CCW in soybean.

### Experiments designed for evaluation of antixenosis against CCW

The IRSGP was tested in a randomized complete block design with four replications (Experiment 1), and a protection row was set around the seed nursery tray in the micro-net-room. In order to verify the reliability of the V1TMD method, the five high resistant (PI227687, JAPAN, HPXQD, AGH and Lamar) and four high susceptible (MYBMD, JLNMH, NN89-29 and DPDLH) in a total of 9 accessions based on the preliminary study [10, 11] were organized into a randomized complete block design experiment with eight replications (Experiment 2). All experiments were conducted in an insecticide-free micro-net-room in greenhouse in Nanjing Agriculture University. Both the two experiments were conducted two times (Test 1 and Test 2), with Test 1 planted in 32 hole seed nursery trays on 18 March 2016 (temperature at 11–21 °C) and Test 2 in same kind of trays on 17 April 2016 (temperature at 15–25 °C), respectively.

### Genotyping the IRSGP with PAV markers

The 76 accessions of the IRSGP were used. The fresh leaves were collected and ground in liquid nitrogen and DNA was extracted with the CTAB method with minor modifications [32]. Komatsu et al. [23] reported that the SSR markers Satt220 and Satt175 were flanking *CCW*-*1* and Satt567 and Satt463 were flanking *CCW*-*2* on linkage group M, respectively, which are located in the physical regions of 9,908,974–15,394,181 bp and 4,559,651–8,283,287 bp, respectively (Wm82.a2 Genomic Sequence of http://soybase.org/). While the PAV markers Gm07PAV0595 and Gm07PAV0389 to be used in the present study are located at 9,453,100 and 6,445,584 bp, respectively [28]. Thus the two PAV markers Gm07PAV0595 and Gm07PAV0389 are tightly linked with *CCW*-*1* and *CCW*-*2*, respectively, therefore were chosen for genotyping the IRSGP. The procedures of PAV marker analysis can refer to Wang et al. [28].

### Statistical procedures

The descriptive statistics of the DLP data, including frequency distribution, mean, *F* and coefficient of variation (*CV*) were obtained using the PROC GLM procedure of SAS program. The heritability in a single test was estimated as *h* ^2^ = *σ*_g_^2^/(*σ*_g_^2^ + *σ*_e_^2^/*r*), where *h* ^2^ = heritability, *σ*_g_^2^ = genotypic variance, *σ*_e_^2^ = error variance, and *r* = the number of replications [33]. The genetic coefficient of variation (*GCV*) was calculated as *GCV* = *σ*_g_/*μ*, where *μ* is the population mean. All the parameters were estimated from the expected mean squares in ANOVA. The efficiency of the V1TMD method and that of the previous artificial infestation method for antixenosis of adult plant against CCW in net-room were compared based on a 1000 plots scale [10, 13].

## Results

### Dynamic evaluation of antixenosis against CCW in IRSGP

Table 1 showed that the average DLP of all accessions increased gradually, as a dynamic process, from 14 to 95% and 15 to 87% in Test 1 and Test 2 of IRSGP (Experiment 1), respectively. The two Tests were conducted in different time (March and April, respectively) under different temperature schemes, therefore, the insects and plants developed faster and the DLP increased faster in the second Test.

Along with the evaluation date moved up and the DLP increased, the *F*-value and *h* ^2^ increased, while the genotypic coefficient of variation (*GCV*) and error coefficient of variation (*CV*) decreased gradually (Table 1). It indicates that the evaluation precision for antixenosis is higher in later dates, but the relative genetic variation among accessions is lower in later dates. Especially, when the test going to the end, the most of the accessions were badly damaged and the average DLP reached more than 80%, in this situation, the highly resistant accessions could be easily identified. While at the early date, when the average DLP of whole accessions was small, such as less than 35%, the highly susceptible accessions could be easily identified. Therefore, the best evaluation dates for distinguish the differences among accessions or for identifying both resistant and susceptible accessions should be decided after balancing the two aspects. Table 1 showed that when the average DLP of all accessions was 50–70% in the middle term of testing stages, the DLP of whole accessions performed a normal-like distribution and should be the best dates for identifying both resistant and susceptible accessions. Here in Table 1, the best dates for antixenosis evaluation were those in italics, i.e. the date of 10th–12th in Test 1 (lower temperature) and 6th–8th in Test 2 (higher temperature). In addition, if the extremely susceptible and extremely resistant accessions are interested, the results in early dates and later dates could be referred respectively in addition to those based on the mid-term results. In the present two tests, at the end only the DLP of PI227687 and Lamar were less than 40%, which were the commonly recognized most resistant accessions up to date [5, 30].

### Stability, precision and accuracy of the novel V1TMD method

The correlation coefficient among evaluation dates and between the two Tests was used to indicate the stability of the antixenosis observations for the novel V1TMD method (Table 2). All the correlation coefficients among evaluation dates within and between the two Tests were significant at 0.01 level, with those within each Test ranging in 0.41–0.91 and 0.55–0.93, respectively, and those between the averages (DLP1 and DLP2) and the individual evaluation dates in two Tests ranging in 0.76–0.94 and 0.73–0.97, respectively. The latter set of correlations (between the averages and individual dates) were high, higher than those of the former set (between individuals), which indicated that the average DLP for all survey dates could provide a relatively stable evaluation of the antixenosis (DLP). Further more, the correlation of DLP1 at DLP1-11 and that of DLP2 at DLP2-7 were respectively 0.94 and 0.97, the highest among different dates, which further confirmed that the middle term testing stage of DLP1-11 and DLP2-7 was most appropriate or the optimal date for antixenosis evaluation. The correlation between DLP1 and DLP2 and between DLP1-11 (the middle term testing stage in Test 1) and DLP2-7 (the middle term testing stage in Test 2) were 0.76 and 0.64, respectively, indicating a relatively high stability between different test times for the novel V1TMD method.

**Table 2:** Correlation of DLP among evaluation dates of the two Tests

Indicator and date	DLP1-3	DLP1-5	DLP1-7	DLP1-9	DLP1-11	DLP1-13	DLP1-15	DLP1	DLP2-3	DLP2-5	DLP2-7	DLP2-9	DLP2-11
DLP1-5	0.85**												
DLP1-7	0.74**	0.88**											
DLP1-9	0.72**	0.81**	0.91**										
DLP1-11	0.66**	0.80**	0.88**	0.90**									
DLP1-13	0.55**	0.63**	0.74**	0.78**	0.81**								
DLP1-15	0.41**	0.48**	0.58**	0.63**	0.68**	0.88**							
DLP1	0.78**	0.88**	0.94**	0.94**	0.94**	0.88**	0.76**						
DLP2-3	0.44**	0.47**	0.54**	0.56**	0.51**	0.45**	0.40**	0.54**					
DLP2-5	0.55**	0.59**	0.67**	0.67**	0.63**	0.53**	0.50**	0.67**	0.75**				
DLP2-7	0.56**	0.61**	0.67**	0.67**	0.64**	0.61**	0.58**	0.70**	0.65**	0.86**			
DLP2-9	0.53**	0.58**	0.65**	0.66**	0.63**	0.68**	0.68**	0.71**	0.60**	0.74**	0.93**		
DLP2-11	0.51**	0.56**	0.62**	0.65**	0.63**	0.75**	0.81**	0.73**	0.55**	0.65**	0.81**	0.91**	
DLP2	0.59**	0.64**	0.72**	0.72**	0.68**	0.67**	0.65**	0.76**	0.73**	0.89**	0.97**	0.95**	0.88**

In “Indicator and date” column, “DLP” represents damaged leaf percentage, the number before “-” indicates the test serial and the number after “-” indicates the days after artificial infestation, for example, “DLP1-3” represents DLP after 3 days of infestation in Test 1. “DLP1” and “DLP2” represent the average DLP over dates of Test 1 and Test 2, respectively

** Represents significance at 0.01 probability level

In order to further demonstrate the stability of the novel V1TMD method, the data from Experiment 1 (IRSGP) and Experiment 2 (9 highly resistant versus susceptible selected accessions) were analyzed for joint analysis of variance, respectively. Table 3 showed that the variances of Accession and Test × Accession interaction were all very significant, and the former was 4 and 59 times of the latter in the two experiments, indicating a good stability of the V1TMD method in evaluating the DLP (antixenosis against CCW).

**Table 3:** Joint ANOVA of DLP at the middle-term testing stage of the two experiments

Source	Experiment 1				Experiment 2			
	*DF*	*SS*	*MS*	*F*	*DF*	*SS*	*MS*	*F*
Accession	75	143,523.9	1913.7	12.8**	8	135,436.3	16,929.5	302.7**
Test	1	135.9	135.9	0.9	1	80.6	80.6	1.4
Test × Accession	75	34,217.6	456.2	3.0**	8	2408.3	301.0	5.4**
Block (Test)	6	6681.3	1113.6	7.4**	14	1805.2	128.9	2.3**
Error	444	66,533.3	149.8		102	5704.6	55.9	

** Represents significance at 0.01 probability level

The precision of the V1TMD method can be examined using the relative size of experiment errors. Smaller error coefficient of variation (*CV*) in the middle and late term testing stage indicates a better precision of the V1TMD method (Table 1). Error coefficient of variation from joint analysis of variance for Experiment 1 and Experiment 2 is 20.7 and 13.3, respectively, which are relatively smaller than or similar to those of the previous method [10, 13], indicating a good precision of the new V1TMD method in evaluating the DLP.

Since multiple tests can be done in a year due to shorter test period of the V1TMD method, the precision and accuracy of the average results from several V1TMD tests in a certain period should be better than that of a single long term test in a same testing period. Even for a single V1TMD test, the significant correlation (*r* = 0.56**) between the V1TMD method for seedling antixenosis in micro-net-room and the previous method for adult plant antixenosis against CCW in large net-room [10] was observed, which indicates the results on young seedlings is relatively consistent to those in adult stages. The consistency is especially true for highly resistant and highly susceptible accessions. In Experiment 1, the 10 most resistant and 10 most susceptible accessions were chosen and listed in Table 4. Among them, the resistant germplasm PI227687, JAPAN, AGH, ZD14, HPXQD, NN89-30 and the susceptible germplasm JNLSD, MYBMD, DPDLH, NN89-29, JLNMH, CYHZM were consistent with Zhan’s and Wu’s selected accessions [10, 11]. In addition, under eight replication conditions in Experiment 2, the distinctness between the resistant accessions Lamar, PI227687, JAPAN, AGH, HPXQD and susceptible accessions MYBMD, DPDLH, NN89-29, JLNMH performed more obviously using V1TMD method. Among these accessions, PI227687 and Lamar were reported with resistance to several leaf-feeding insects [5, 30]. The same high resistant and high susceptible accessions were screened out by different methods, indicating that the novel V1TMD method was accurate enough. Accordingly, the above highly resistant and susceptible accessions were selected as the standard checks for the V1TMD method.

**Table 4:** The high resistant and susceptible accessions screened out

Accession name	DLP (%)							LW (g)
	Experiment 1			Experiment 2			Zhan [10]	Wu [11]
	DLP1	DLP2	Mean 1	DLP1	DLP2	Mean 2		
Resistant								
Lamar	12	5	9	12	7	9		
*PI227687*	20	9	15	23	13	18	23	0.209
*JAPAN*	32	20	26	35	21	28	18	0.258
P64	39	36	38					
Bethol	40	30	35					
*AGH*	43	45	44	54	35	44	20	0.240
*ZD14*	40	50	45				18	0.347
TSBPHDJ	44	46	45				23	
*HPXQD*	40	52	46	50	46	48	16	0.230
*NN89-30*	50	47	49				21	0.304
Average 1	36	34	35	35	24	29	20	0.265
Susceptible								
*JNLSD*	73	83	78				38	0.650
*MYBMD*	71	78	75	83	85	84	34	0.733
XJ2	64	83	74					
*DPDLH*	62	77	70	72	79	75	46	0.747
JXQDA	65	74	70				47	
XTDD	62	73	68					
*NN89-29*	54	75	65	73	76	74	42	0.659
FJ341	64	63	64				36	
*JLNMH*	54	69	62	64	70	67	49	0.702
*CYHZM*	61	61	61				42	0.671
Average 2	63	74	69	73	78	75	42	0.693

In “Accessions name” column, accessions in italics are the same with the Zhan’s and Wu’s results [10, 11]. Average 1 and Average 2 are the mean of resistant and susceptible accessions, respectively. “DLP1” and “DLP2” represent the average DLP over dates of Test 1 and Test 2, respectively. Mean 1 and Mean 2 are the mean of Experiment 1 and Experiment 2, respectively

### The V1TMD results confirmed by PAV markers of *CCW*-*1* and *CCW*-*2* in IRSGP

**Fig. 3:**
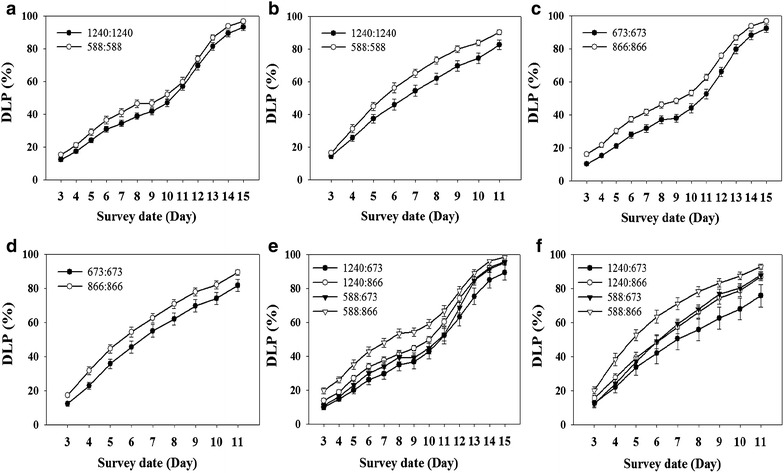
Allelic effects of PAV markers Gm07PAV0595 and Gm07PAV0389 flanking *CCW*-*1* and *CCW*-*2*, respectively, on different survey dates. **a**, **b** Allelic effects of the single PAV marker Gm07PAV0595 at different survey dates in Test 1 and Test 2 of Experiment 1, respectively. 1240 and 588 are the allele size of resistance allele and susceptibility allele, respectively. **c**, **d** Allelic effects of the single PAV marker Gm07PAV0389 at different survey dates in Test 1 and Test 2 of Experiment 1, respectively. 673 and 866 are the allele size of resistance allele and susceptibility allele, respectively. **e**, **f** Allelic effects of the flanking two PAV markers Gm07PAV0595 and Gm07PAV0389 at different survey dates in the two Tests of Experiments 1, respectively. 1240:673 represents the combination of both resistance allele, and 1240:866 represents the combination of resistance allele at *CCW*-*1* and susceptible allele at *CCW*-*2*, and 588:673 represents the combination of susceptible allele at *CCW*-*1* and resistance allele at *CCW*-*2*, and 588:866 represents the combination of both susceptible allele

The PAV markers Gm07PAV0595 and Gm07PAV0389 are closely linked to the identified QTL *CCW*-*1* and *CCW*-*2*, respectively [23, 28]. If the evaluation of antixenosis (DLP value) using the V1TMD method is correct, the genotypes with resistance allele and those with susceptible allele should have their DLP values constantly different through all the observing dates. Figure 3a, b showed the DLP of accessions with the resistance allele “1240” on the locus of Gm07PAV0595 (possessed by resistance accessions, such as Lamar and PI227687) was lower than that of the other group of accessions with susceptible allele “588” on the same locus (possessed by susceptible accessions, such as MYBMD and DPDLH). This distinction held for all the observation dates in both Test 1 and Test 2 of Experiment 1. So the two growth lines of the two allele types obviously separated from each other on all the dates. Similarly, the DLP of accessions with the resistance allele “673” on the locus of Gm07PAV0389 (possessed by Lamar and PI227687) was lower than that of the other group of accessions with susceptible allele “866” on the same locus (possessed by MYBMD, DPDLH). This distinction held also for all the observation dates in both Test 1 and Test 2 of Experiment 1 (Fig. 3c, d). As the recombination of the two loci is concerned, there are four kinds of alleles. Figure 3e, f showed that the distinction of DLP values among the four kinds of alleles held for all the observation dates in both Test 1 and 2 in the IRSGP population. It is obvious that the four growth lines separated from each other. Thus, the DLP evaluation from the novel V1TMD method for antixenosis against CCW is relatively correct and relevant.

### Efficiency of the V1TMD method compared to the previous method

As it has been indicated above, the previous evaluation method for antixenosis against CCW in soybean was conducted usually on adult plants in large net room for almost a full growth season, therefore, needed much more room, manpower, and spare expenses. Even so, the precision and accuracy are not coincided with the expenses; sometimes the experiment error can not be well-controlled due to the net-room conditions. Table 5 shows the empirical estimation on the efficiency of the novel V1TMD method compared with the previous method for evaluation of antixenosis against CCW in soybean. Here the following six items were taken into consideration: labor consumption, number of larvae needed, survey time consumed, length of a test cycle, net-room size and estimated cost. As the estimated cost is concerned, the efficiency of the novel V1TMD method is about 12 times of that of the previous method. A more important merit of the V1TMD method is that its test cycle can be seriously shortened so multiple tests can be conducted in a year.

**Table 5:** Efficiency of V1TMD method compared with the previous method for evaluation of antixenosis against CCW

Item	Previous method	V1TMD method	Efficiency ratio
Labor (person-month)	12	1	12
Number of larvae (head)	12,000	2000	6
Survey time (h)	20	5	4
Test cycle (month)	12	1	12
Netroom area (m^2^)	560	4.7	119.1
Cost (rent, labor wages and materials, ¥)	12,600	1000	12.6

Based on testing 1000 plots

## Discussion

### V1TMD is a high-throughput phenotyping procedure

Araus and Cairns [34] indicated that the phenotyping of the appropriate traits, using low cost, easy-to-handle tools, should become an integral and key component in the breeding procedure. Haghighattalab et al. [35] also emphasized that the high-throughput phenotyping platforms could provide the keys to connecting the genotype to phenotype by both increasing the capacity and precision and reducing the time to evaluate huge germplasm populations. In spite of the low throughput phenotyping property of the previous methods in evaluation of antixenosis against CCW, the novel V1TMD method can treat large scale evaluation of antixenosis in germplasm study and breeding programs due to the shorter growth time of soybean seedling, fewer leaves, higher larval survival rate and less artificial infestation times, thus the large scale experiment could be carried out quickly and smoothly. Thus the V1TMD method is in fact a high-throughput phenotyping procedure in evaluation of antixenosis against CCW in soybean, and it can match the modern high throughput genotyping for germplasm and plant breeding studies. In addition, the V1TMD method is suitable for a dynamic observation of the development process of antixenosis against CCW in a population, therefore, partial distributions at serial dates can be observed easily among which the best date for population frequency distribution can be chosen.

### V1TMD is potential in raising evaluation accuracy and precision of antixenosis against CCW

In addition to the high efficiency, more advantages of the V1TMD method are interested to the researchers. Compared to the previous method of antixenosis bioassay in net-room, the V1TMD method puts the experiment in a micro-net-room which in turn in a greenhouse, the environment, including biological and non-biological factors, can be well-controlled. That means in a very small area of the micro-net-room, the environment conditions can be controlled uniformly, while under the double-controlled rooms, the foreign insects and pathogens are excluded, therefore, the accuracy and precision of the V1TMD method can be raised. Especially the soybean seedlings are infested with third-instar larvae chosen to have visually uniform size and the inoculated CCW larvae may move freely within a short distance to pick up their preferred accessions for feeding. In addition, the plants at the young seedling stage usually have a small leaf size and a same number of leaves/leaflets, which fits an accurate and precise evaluation. In the present study, the fact that the survival rate of the insects kept alive more than 50% at the end, much more than those obtained from the previous method, demonstrated the evaluation was under active conditions for the insects, therefore, the V1TMD method could provide a higher accuracy and precision.

### Utilization of the V1TMD method and further consideration

It is sure that the high-throughput phenotyping method of antixenosis evaluation V1TMD fits a large scale test, therefore fits germplasm study and breeding for antixenosis against CCW in soybean. Especially it fits early generation testing for the trait in breeding programs. This method does not necessary fit the antixenosis against CCW only, it can be extended to other leaf-feeding insects on soybean, as well as other crops. In addition, in utilization of the V1TMD method, the accurate sensory evaluation of DLP is the key. As indicated above, the observer should be skilled and work through the full experiment, at least a replication. Now digital photography and remote sensing are commonly recognized promising approaches in phenotyping the traits [36]. If the facility of image analysis method can be used to measure the 3D distribution of DLP in a large scale experiment, the novel V1TMD method of antixenosis to CCW can be further accelerated and improved.

For a broad utilization of the V1TMD method, further studies are needed. For example, when the seedlings were used, the assumption was the results from the seedlings could represent those at the adult or later stage. From our experience, it is basically true, but we are not sure whether it fits a broad spectrum of materials, which is to be further answered. Moreover, if the micro-net-room is put in an air-conditioned green house with the day length and temperature controlled, the V1TMD evaluation results from different months or seasons can be compared directly. But if the greenhouse is not air-conditioned or the environment is not well-controlled, how can make the obtained results from different months or seasons comparable? Can the use of common checks be effective? These are also to be further studied.

## Conclusion

A dynamic and efficient evaluation procedure for antixenosis against CCW characterized with using V1 stage soybean seedlings infested with third-instar larvae in a micro-net-room in greenhouse with damaged leaf percentage (DLP) as indicator was established and designated V1TMD method. The highly resistant accessions Lamar, PI227687, JAPAN, AGH, HPXQD and highly susceptible accessions JNLSD, MYBMD, DPDLH, NN89-29 and JLNMH are used as the standard checks and the average DLP over all observation dates in a test is an optimal indicator while the middle term testing stage is the optimal time for DLP evaluation. The stability, precision and accuracy were demonstrated to be better than the previous method using adult plant tested in net-room and the efficiency was estimated to be 12 times of the previous method.

## Additional file

**Additional file 1: Table S1.** The DLPs of all accessions at all evaluation dates in Test 1 and Test 2 of Experiment 1.

## Abbreviations

CCW: common cutworm
DLP: damaged leaf percentage
IRSGP: insect-resistant versus -susceptible germplasm population
PAV: presence/absence variation
V1TMD: the procedure for evaluation of antixenosis against common cutworm characterized with using V1 stage soybean seedlings infested with third-instar larvae in a micro-net-room in greenhouse with damaged leaf percentage as indicator
V1: first trifoliolate
VE: emergence stage
VC: cotyledon stage
